# Anti-Nuclear Antibodies Patterns in Patients With Systemic Lupus Erythematosus and Their Correlation With Other Diagnostic Immunological Parameters

**DOI:** 10.3389/fimmu.2022.850759

**Published:** 2022-03-14

**Authors:** Jamil A. Al-Mughales

**Affiliations:** ^1^Department of Medical Microbiology and Parasitology, Faculty of Medicine, King Abdulaziz University, Jeddah, Saudi Arabia; ^2^Department of Clinical Laboratory Medicine, Diagnostic Immunology Division, King Abdulaziz University Hospital, Jeddah, Saudi Arabia

**Keywords:** antinuclear antibodies, ANA, patterns, systemic lupus erythematosus, SlE, immunofluorescence, anti-dsDNA

## Abstract

**Background:**

Antinuclear antibodies (ANA) are major immunodiagnostic tools in systemic lupus erythematosus (SLE); however, their clinical and pathogenic roles are not yet elucidated and are a subject of controversy.

**Objectives:**

The aim of the study is to explore the pathogenic significance of ANA patterns among SLE patients, by analyzing their association with ANA titers, complement levels and other pathogenic immune markers, namely, anti-double-stranded DNA (anti-dsDNA), complements C3 and C4, rheumatoid factor (RF), anticardiolipin antibodies IgG (ACL IgG) and IgM (ACL IgM), Beta-2 Glycoprotein 1 Antibodies (β2-GP) IgG (β2-IgM) and IgM (β2-IgM), and lupus anticoagulant (LA).

**Method:**

A comparative cross-sectional study was conducted among 495 SLE patients, who were diagnosed and classified by consultant rheumatologists according to the new European League Against Rheumatism (EULAR)/American College of Rheumatology (ACR) 2019 criteria. SLE immunodiagnostic profiles were analyzed including the following parameters: ANA antibody titers and staining patterns, anti-dsDNA, C3 and C4 levels, aCL, and anti-β2-GP and LA.

**Result:**

The most frequently observed ANA patterns were the speckled (52.1%) and homogeneous (35.2%) patterns, while other patterns were rare representing less than 7% of the patients each. ANA titers were highest in patients with mixed pattern followed by the speckled pattern. Of all the investigated patterns, the peripheral pattern showed the most pathogenic immune profile, namely, highest levels of anti-dsDNA, lowest levels of C4, and highest levels of aCL and β2-GP IgG and IgM.

**Conclusion:**

This retrospective study showed that speckled followed by homogeneous ANA patterns were predominant accounting for 52.1 and 35.2% of the patients. The ANA pattern showed several associations with other immune markers that are documented to have significant clinical implications in SLE. Peripheral, mixed, and speckled patterns were associated with higher profiles of immune markers indicative of a potential prognostic value of these patterns in SLE.

## 1 Introduction

Systemic lupus erythematosus (SLE) is a systemic autoimmune disease characterized by flare up phases and others with low disease activity (1). It affects multiple organs such as serous membranes, renal, nervous, and cardiovascular systems, and joints and skin, resulting in multiorgan damage. Among the challenging aspects of SLE are its enigmatic pathophysiology and extremely variable clinical presentations and manifestations, both between patients and within the same patient over time (2–4). Further, more than 180 different self-antigens were discovered to bind autoantibodies in SLE patients, with high heterogeneity and variable expressions between the patients. These autoantibodies mainly target intracellular components in the nucleus, such as single- (ssDNA) and double-stranded (dsDNA) DNA, and histones, and are hence called antinuclear antibodies (ANA) (5–7). Such immunological profile brings an odd complexity in understanding the pathophysiology of the disease. On the other hand, most of these antibodies are not specific for SLE. ANA can be seen in all kinds of rheumatic diseases (8). ANA, anti-dsDNA, phospholipids are included in the 11 criteria to diagnose SLE including the new European League Against Rheumatism (EULAR)/American College of Rheumatology (ACR) 2019 classification criteria which has a sensitivity of 96% and a specificity of 93.4% (9). Because high concentrations of anti-ds-DNA antibodies are almost exclusively present in SLE patients, anti-ds-DNA antibodies are more SLE-specific (10). Besides, ANAs titers and antigenic target are predictive of the disease pathogenicity and prognosis. Most specifically, anti-ds DNAs titers have a diagnostic value in indicating SLE activity along with the level of organ involvement (11–14).

Despite being part of the EULAR/ACR criteria, the clinical utility of ANA and anti-DNA assays in SLE patients is highly debated due to their inconsistency and non-resolution of their pathogenic roles (12, 15–18). On the other hand, technical challenges of the assays impact their interpretability, notably concerning the immunofluorescence staining patterns of ANA, whose pathogenic role is highly controversial (19–21). In the present study, we aimed to further explore the pathogenic significance of ANA patterns among patients with SLE, by analyzing their association with ANA titers, complement levels and other pathogenic immune markers, namely, complement C3 and C4, rheumatoid factor (RF), anticardiolipin antibodies IgG (ACL IgG) and IgM (ACL IgM), Beta-2 Glycoprotein 1 Antibodies IgG (β2-IgM) and IgM (β2-IgM), and lupus anticoagulant (LA). Such correlations would contribute to the pathogenic or prognostic significance of ANA patterns in SLE.

## 2 Methods

### 2.1 Design and Participants

This was a cross-sectional study conducted at the Immunodiagnostic unit of the Microbiology and Parasitology Department of the King Abdulaziz University Hospital, which is a referral immunodiagnostic center in Jeddah, Saudi Arabia. The study was ethically approved by the institutional review board of the King Abdulziz University (Ref. No. 130-21).

### 2.2 Participants

The study involved patients from all age groups diagnosed and classified SLE by a consultant rheumatologist and followed in the participating center from January 2018 to December 2020. Cases were diagnosed and defined in accordance with the EULAR/ACR criteria 2019 (22). Patients having no results for ANA pattern were excluded. A convenience sampling was used to include all consecutive patients that fulfilled the eligibility criteria.

### 2.3 Data Collection

#### 2.3.1 Demographic Data of Patients

The age, gender and nationality of patients were collected from the electronic files of the patient.

#### 2.3.2 Immune Assays

SLE immunodiagnostic profiles were analyzed including the following parameters: ANA antibody titers and staining patterns, anti-dsDNA antibodies, Complements levels (C3 and C4), anticardiolipin (aCL), anti-b2 glycoprotein, and lupus anticoagulant antibodies.

ANA tests were performed by indirect immunofluorescence (IIF) technique utilizing human epithelial cells (Hep-2) fixed on glass slides which were commercially prepared (Aesku Diagnostics; Windlesham; Germany). Briefly, the sera of patients were diluted with phosphate-buffered saline (PBS) and were overplayed in a well on the Hep-2 substrate slide. The slides were placed in a humid chamber, incubated for 20 min at room temperature, and was followed by washing over 10 min in two changes of PBS on a reciprocating shaker. The substrate was then covered with approximately one drop of the conjugate solution. After 30 min of incubation at room temperature, the slides were washed in PBS and were immediately covered with glycerol-PBS (mounting medium) and viewed with a standard immunofluorescence microscope (Olympus, Japan). The fluorescence strength depends on sample titration, based on 1:40 dilutions. Zero titer referred to absence of ANA on immunofluorescence (ANA negative). ANA antibody patterns were described to be as peripheral, speckled, homogenous, nucleolar, and centromere patterns. Antibodies to dsDNA were performed by the enzyme linked immunosorbent assay (ELISA) technique using the same INOVA System Quanta Lite™ Ds-DNA Kit. Briefly, the sera of the patients were diluted with an ELISA sample diluent and added to separate wells of micro well plate. The strips were covered and incubated for 30 min at room temperature. Then the conjugate was added to each well and incubated for 30 min and washed. Then the substrate was added to each well and incubated for 30 min at room temperature. An ELISA stopping solution was added to each well, and the plates were read at 450 nm using an ELISA reader (Dyntech, USA). Anti-b2 glycoproteins antibodies were performed by Alegria instrument (Ogentec; Mainz; Germany). The instrument used the ELISA principle mentioned above. The BNII nephelometry instrument (Semen’s; Germany) was used for C3, C4, and C-Reactive Protein (CRP) measurements. Lupus anti-coagulant was measured according to the commercial insert (Diagnostica Stago, Asnières-sur-Seine, France). Bechman coulter (USA) was used to measure absolute cells counts.

All immunodiagnostic analyses were performed at the same laboratory and used the same methods for all patients.

#### 2.3.3 Other Biological Parameters

In addition, CRP level, absolute leucocytes, neutrophils count, and hemoglobin levels have been included in the analysis, and also prothrombin time (PT) and partial thromboplastin time (PTT). Assays were performed in accordance with the standard laboratory methods and in compliance with respective guidelines of the manufacturers (Diagnostica Stago, Asnières-sur-Seine, France).

### 2.4 Variables

Primary outcome of the study was the ANA pattern, according to which patients were classified for all inferential analyses. The other variables of interest, namely, age, gender, nationality, ethnic group, CRP, PTT, neutrophils and lymphocytes counts, ANA titers, C3, C4, RF, ACL, β2, and LA were analyzed as the independent variables.

### 2.5 Statistical Methods

Data was analyzed using the statistical package for the social sciences (SPSS), version 21 for Windows. Categorical variables were presented as frequency and percentage. Scale variables were presented as means and standard deviation or median and interquartile range (IQR), as applicable. Chi square and Fisher’s exact test were used to analyze the associations between the categorical variables. The nonparametric ranked, Kruskal–Wallis test was used to analyze the association of ANA pattern with ordinal variables such as ANA titer, or non-normally distributed scale variables. OneWay Analysis of Variance (ANOVA) was used to analyze the variance of scale variables by ANA pattern; significant results underwent *post hoc* analysis using Tukey’s honestly significant difference (HSD) test. Significance level was set for at p <0.05.

## 3 Results

### 3.1 Characteristics of Participants

Out of 495 participants, 88.1% were women. The mean (SD) age was 36.81 (15.18) years while the median (IQR) age was 35 (18) years. Regarding nationality and ethnicity, majority of the participants were of Arabian descent (70.7%), of Saudi (57.0%) or Yemeni (13.7%) nationality. Other ethnic groups such as Middle-Eastern, African or Asian were a minority (**Table 1**).

**Table 1:** Participants’ demographic characteristics and nonspecific biological markers.

Parameter/Category	Frequency	Percentage	Mean (range)	SD
**Demographic data**				
**Age (years)**			36.81 (8–92)	15.18
**Gender**				
Male	59	11.9		
Female	436	88.1		
**Nationality**				
Saudi	282	57.0		
Yemeni	68	13.7		
Chadian	27	5.5		
Pakistani	18	3.6		
Sudanese	14	2.8		
Indian	12	2.4		
Palestinian	15	3.0		
Other	59	11.9		
**Ethnic group**				
Arabian tribes	350	70.7		
Middle-Eastern	31	6.3		
Afro-Arab	35	7.1		
African	32	6.5		
South Asian	37	7.5		
Southeast Asian	10	2.0		

### 3.2 Non-Specific and Specific Biological Markers

Data on non-specific biological markers were available for 75.1–98.2% of the patients, depending on the marker. These showed positive CRP (31.6%) and low and high PT (0.3 and 37.4%) and PTT (6.7 and 21.4%), respectively. Remarkably, hemoglobin was relatively low with a median 11.6 g/dl. Specific immune markers were available for 53.7–99.6%, depending on the marker. Anti-dsDNA was high for all patients, with a median value of 517.9 IU/ml, and lupus anticoagulant was positive (>8 s) for 99.4% of the tested patients. C3 and C4 complement showed median values of 0.89 and 0.17 IU/ml, respectively, while rheumatoid factor was positive for 12.8% of the patients. ACL antibodies were weak positive or positive in approximately 1 out of 8 patients including IgG (6.1 and 6.9%) and IgM (8.8%and 2.1%). On the other hand, β2-glycoprotein antibodies including IgG and IgM were weak positive (11.4 and 4.9%) and positive or strongly positive (10.3 and 3.9%), respectively (**Table 2**).

**Table 2:** Participants’ specific and non-specific biological markers.

Marker / Level	N	Frequency	Percentage	Median	Q1, Q3
**Non-specific markers**					
**CRP**	**478**			3.74	3.16, 13.2
Negative (<10)		327	68.4		
Positive (10+)		151	31.6		
* Moderate (10–<20)*		*82*	*16.1*		
* Frank (20–<100)*		*42*	*9.8*		
* High (100+)*		*26*	*5.6*		
Not done	17				
**Prothrombin time (s)**	**372**			11.90	10.5, 13.2
Low (<9.4)		1	0.3		
Normal (9.4–12.5 s)		232	62.4		
High (>12.5)		139	37.4		
Not done	123				
**PTT (s)**	**374**			31.4	25.7, 35.8
Low (<25)		25	6.7		
Normal (25–37 s)		269	71.9		
High (>37)		80	21.4		
Not done	121				
** Neutrophils**	**482**			3	2, 5
** Lymphocytes**	**481**			2	1, 3
** Hemoglobin (g/dl)**	**486**			11.6	10.4, 12.6
**Specific markers**					
** ADNA**	**493**			517.9	315.7, 755.6
** C3**	**485**			0.89	0.67, 1.11
** C4**	**485**			0.17	0.10, 0.24
**Rheumatoid factor**	**266**			11.0	10.1, 11.5
Negative (<15 IU/ml)		232	87.2		
Positive (>15 IU/ml)		34	12.8		
**ACL IgG**	**376**				
Negative (<15 IU/ml)		327	87.0		
Weak positive (15–40 IU/ml)		23	6.1		
Positive (>40 IU/ml)		26	6.9		
**ACL IgM**	**386**				
Negative (<15 IU/ml)		344	89.1		
Weak positive (15–40 IU/ml)		34	8.8		
Positive (>40 IU/ml)		8	2.1		
**β2-IgG**	**271**				
Negative (<15 U/ml)		212	78.2		
Weak positive (15–<40 U/ml)		31	11.4		
Positive (40–<80 U/ml)		9	3.3		
Strongly positive (80+ U/ml)		19	7.0		
**β2-IgM**	**285**				
Negative (<15 U/ml)		260	91.2		
Weak positive (15–<40 U/ml)		14	4.9		
Positive (40–<80 U/ml)		9	3.2		
Strongly positive (80+ U/ml)		2	0.7		
**Lupus anticoagulant**	**344**				
Normal (0–45 s)		272	79.1		
High (>45 s)		72	20.9		

ADNA, Anti-double stranded DNA; ACL, anticardiolipin; Beta-2 Glycoprotein 1 Antibodies, IgG and IgM.

Bold values correspond to the total of patients with available data in the given parameter.

### 3.3 Patterns of Antinuclear Antibodies and the Correlated Titers

The most frequently observed ANA patterns were the speckled (52.1%) and homogeneous (35.2%) patterns; while other patterns were rare representing less than 7% of the patients each. Titers of ANA were 1:640 or higher in 82.4% of the patients. Titers were highest in patients with mixed pattern followed by peripheral and speckled patterns, where 81.3, 80, and 70.9% of the patients had ANA titer 1:1280 respectively, and the results were statistically significant using both chi square (p <0.001) and nonparametric tests (p <0.001) (**Table 3**).

**Table 3:** Titers and patterns of antinuclear antibodies in SLE patients.

Titers	Total	Pattern					
		Speckled	Homogenous	Mixed pattern	Nucleolar	Centromere	Peripheral
**Total**	**N (%)**	**258 (52.1)**	**174 (35.2)**	**32 (6.5)**	**18 (3.6)**	**3 (0.6)**	**10 (0.2)**
1:40	**1 (0.2)**	1 (0.4)	0 (0.0)	0 (0.0)	0 (0.0)	0 (0.0)	0 (0.0)
1:80	**1 (0.2)**	0 (0.0)	0 (0.0)	0 (0.0)	1 (5.6)	0 (0.0)	0 (0.0)
1:160	**30 (6.1)**	11 (4.3)	13 (7.5)	2 (6.3)	4 (22.2)	0 (0.0)	0 (0.0)
1:320	**55 (11.1)**	22 (8.5)	28 (16.1)	2 (6.3)	2 (11.1)	1 (33.3)	0 (0.0)
1:640	**89 (18.0)**	41 (15.9)	38 (21.8)	2 (6.3)	6 (33.3)	0 (0.0)	2 (20.0)
1:1280	**319 (64.4)**	183 (70.9)	95 (54.6)	26 (81.3)	5 (27.8)	2 (66.7)	8 (80.0)

Chi square: statistics = 63.75, df = 25, p <0.001.

Kruskal–Wallis test: statistics = 29.09, df = 5, p <0.001. Bold values correspond to the totals in the corresponding pattern or titer level.

### 3.4 Association of ANA Pattern With Demographic Factors

Nucleolar pattern was associated with an age of an older patients (mean = 50.6 years, SD = 17.7) compared with the other patterns (mean age ≤39.2 years) and the result was statistically significant (OneWay ANOVA with Tukey HSD *post hoc*, p = 0.001). No statistical significance was found between the ANA pattern and the ethnic group; however, a homogeneous pattern was relatively predominant in Yemeni (41.2%) patients, while a speckled pattern was predominant in most other nationalities notably in Indian (83.3%), Chadian (74.1%), and Sudanese (71.4%) patients. The previous results were statistically significant (Chi square, p <0.001) (**Table 4**).

**Table 4:** Association of ANA patterns with demographic factors.

Factor/Level	N	Pattern, %						p-value
		Speckled	Homogeneous	Mixed	Nucleolar	Centromere	Peripheral	
**Age**	**495**							
Mean		35.1	37.8	39.2	50.6^§^	34.0	30.1	
SD		13.0	16.6	18.2	17.7	5.2	16.3	.001*
**Gender**								
Male	59	54.2%	39.0%	1.7%	3.4%	0.0%	1.7%	
Female	436	51.8%	34.6%	7.1%	3.7%	0.7%	2.1%	.677
**Nationality**								
Saudi	282	**53.9%**	35.1%	5.0%	3.9%	0.4%	1.8%	
Yemeni	68	35.3%	**41.2%**	11.8%	7.4%	0.0%	4.4%	
Chadian	27	**74.1%**	22.2%	3.7%	0.0%	0.0%	0.0%	
Pakistani	18	**55.6%**	22.2%	11.1%	0.0%	11.1%	0.0%	
Sudanese	14	**71.4%**	21.4%	0.0%	0.0%	0.0%	7.1%	
Indian	12	**83.3%**	16.7%	0.0%	0.0%	0.0%	0.0%	
Palestinian	15	**46.7%**	**46.7%**	6.7%	0.0%	0.0%	0.0%	
Other	59	42.4%	42.4%	10.2%	3.4%	0.0%	1.7%	<0.001*
**Ethnic group**								
Arab tribes	350	**50.3%**	36.3%	6.3%	4.6%	0.3%	2.3%	
Middle-Eastern	31	38.7%	**41.9%**	12.9%	3.2%	0.0%	3.2%	
Afro-Arab	35	**60.0%**	28.6%	8.6%	0.0%	0.0%	2.9%	
African	32	**65.6%**	31.3%	3.1%	0.0%	0.0%	0.0%	
South Asian	37	**59.5%**	29.7%	5.4%	0.0%	5.4%	0.0%	
Southeast Asian	10	**60.0%**	30.0%	0.0%	10.0%	0.0%	0.0%	.174

Percentages are calculated on the row variable and categories.

^§^Value significantly higher compared to speckled and homogeneous in post hoc analysis using Tukey HSD test. *Statistically significant result (p<0.05). Bold values correspond to the most common pattern in the given factor category.

### 3.5 Association of ANA Pattern With Specific and Nonspecific Biological Markers

**Figure 1:**
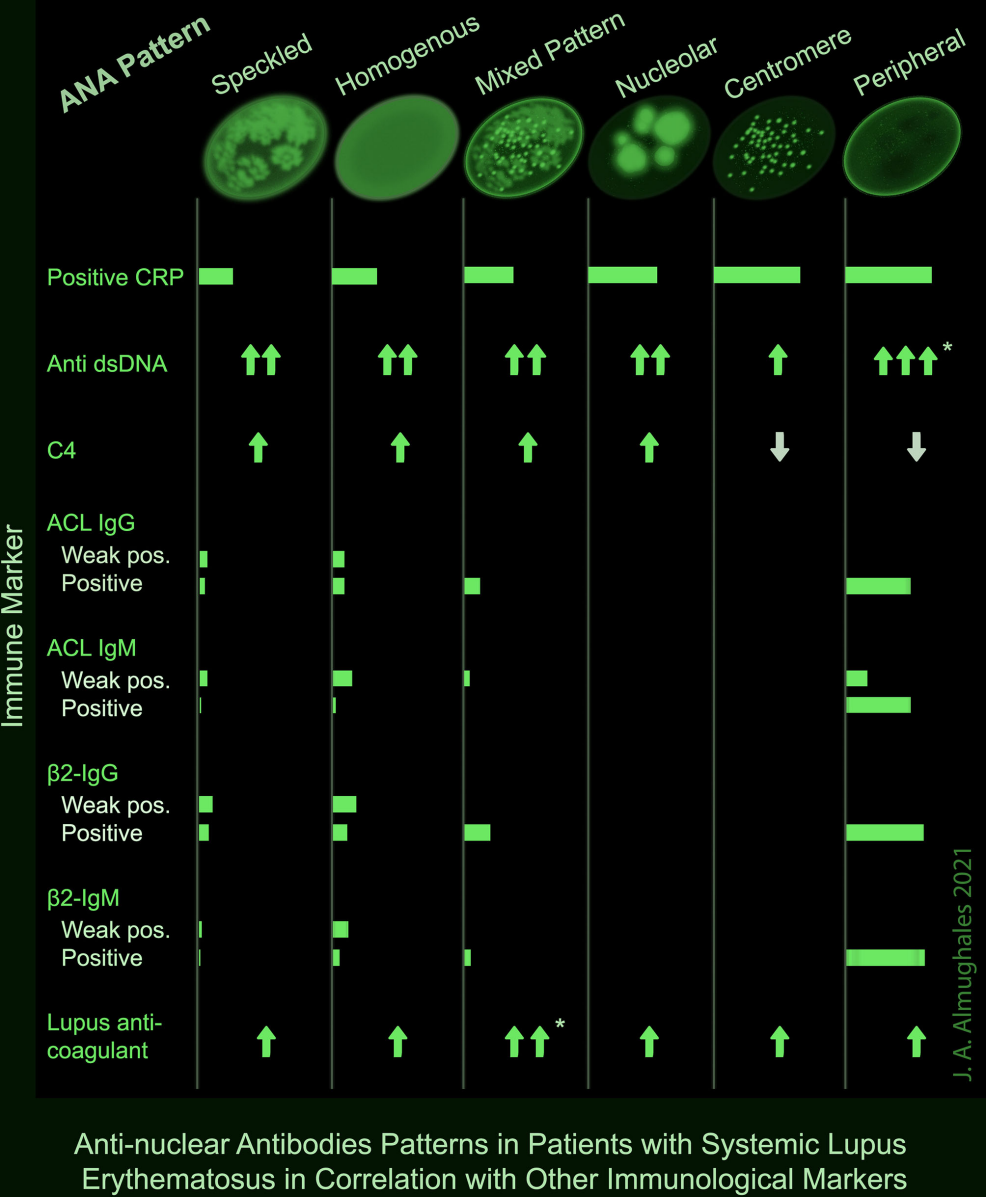
Summary of the significant associations of ANA patterns with other immune markers in patients with SLE. * Significance confirmed by post hoc analysis.

A positive CRP was observed in two-third of patients with centromere or peripheral ANA pattern, and 53.3% of those with nucleolar pattern; while it was lowest (25.6%) in patients with speckled pattern (p = 0.008). Peripheral pattern was also associated with the highest levels of anti-dsDNA (p = 0.007) and mixed pattern was associated with the highest levels of lupus anticoagulant (p = 0.003) as demonstrated both in OneWay ANOVA and Kruskal–Wallis tests and confirmed with *post hoc* analysis. No significant association was found between the ANA pattern and C3; however, C4 was lowest in peripheral and centromere patterns (p = 0.003). ACL antibodies including IgG and IgM were frequently detected in peripheral pattern (50 and 67.7%) including both weak positive and positive results; while they were rarely detected in speckled (10 and 6.9%), homogenous (17.8 and 17.4%) and mixed (12.5 and 4.2%) patterns, respectively, and absent in nucleolar and centromere (p <0.001). Likewise, β2-glycoprotein IgG and IgM antibodies were positive or strongly positive in 60% of the patients with peripheral pattern, compared with up to 20% in speckled, homogenous and mixed patterns and 0% in nucleolar and centromere (p <0.001) (**Table 5**). A summary of the significant associations of ANA patterns with CRP and other immune markers is depicted in a take-home **Figure 1**.

**Table 5:** Association of ANA pattern with specific and nonspecific biological markers.

Factor/Level	Pattern, %						p-value
	Speckled	Homogenous	Mixed	Nucleolar	Centromere	Peripheral	
N	258	174	32	18	3	10	
**CRP**							
Negative	74.4%	65.1%	62.5%	46.7%	33.3%	33.3%	
Positive	25.6%	34.9%	37.5%	53.3%	66.7%	66.7%	.008*
**ADNA (anti-dsDNA)**							
Mean	565.6	576.5	628	474.5	331.3	908.3^‡^	
SD	323.1	297.4	331.1	240.1	143.2	331.1	.007*
**Rheumatoid factor**							
Negative	83.7%	89.7%	94.4%	91.7%	100.0%	100.0%	
Positive	16.3%	10.3%	5.6%	8.3%	0.0%	0.0%	.511
**C3**							
Median	0.87	0.92	0.88	0.97	0.83	0.71	
IQR	0.44	0.44	0.37	0.36	–	0.40	.066^K^
**C4**							
Median	0.15	0.18	0.21	0.18	0.07	0.05	
IQR	0.13	0.14	0.15	0.26	–	0.37	.003* ^K^
**ACL IgG**							
Negative	90.0%	82.3%	87.5%	100.0%	100.0%	50.0%	
Weak positive	5.7%	8.9%	0.0%	0.0%	0.0%	0.0%	
Positive	4.3%	8.9%	12.5%	0.0%	0.0%	50.0%	.003*
**ACL IgM**							
Negative	93.1%	82.7%	95.8%	100.0%	100.0%	33.3%	
Weak positive	6.0%	15.0%	4.2%	0.0%	0.0%	16.7%	
Positive	0.9%	2.4%	0.0%	0.0%	0.0%	50.0%	<.001*
**β2-IgG**							
Negative	82.7%	70.5%	80.0%	100.0%	100.0%	40.0%	
Weak positive	10.0%	18.2%	0.0%	0.0%	0.0%	0.0%	
Positive	5.3%	0.0%	0.0%	0.0%	0.0%	20.0%	
Strongly positive	2.0%	11.4%	20.0%	0.0%	0.0%	40.0%	<.001*
**β2-IgM**							
Negative	96.8%	83.0%	95.0%	100.0%	100.0%	40.0%	
Weak positive	1.9%	11.7%	0.0%	0.0%	0.0%	0.0%	
Positive	1.3%	5.3%	5.0%	0.0%	0.0%	20.0%	
Strongly positive	0.0%	0.0%	0.0%	0.0%	0.0%	40.0%	<.001*
**Prothrombin Time**							
Low	0.5%	0.0%	0.0%	0.0%	0.0%	0.0%	
Normal	63.3%	62.4%	68.4%	61.5%	0.0%	40.0%	
High	36.2%	37.6%	31.6%	38.5%	100.0%	60.0%	.698
**PTT**							
Low	9.3%	3.1%	0.0%	7.7%	0.0%	20.0%	
Normal	70.7%	77.3%	60.0%	76.9%	0.0%	60.0%	
High	20.0%	19.5%	40.0%	15.4%	100.0%	20.0%	.010*
**Lupus anticoagulant**							
Mean	39.98	41.81	56.43^§^	41.83	62.8	39.39	
SD	17.45	13.14	25.43	32.43	40.88	10.07	.003*
Median	36.00	37.15	42.30	32.80	39.20	36.25	
IQR	10.90	11.20	50.00	14.55	–	20.78	.006* ^K^

^§^Significantly higher compared to speckled and homogenous in post hoc analysis using Tukey HSD test.

^‡^Significantly higher compared to speckled, homogenous and nucleolar in post hoc analysis (Tukey HSD test).

ACL, Anticardiolipin antibody; IQR, Interquartile range; PTT, Partial Thromboplastin Time; ^K^, Kruskal–Wallis test; *statistically significant result (p <0.05).

## 4 Discussion

### 4.1 Summary of Findings

The present retrospective study explored the controversial issue of the clinical significance of ANA pattern in SLE patients, and whether some patterns have a diagnostic or predictive value for disease severity. In this cohort of 495 SLE patients with high anti-dsDNA levels and ANA titers, speckled followed by homogeneous ANA patterns were predominant accounting for 52.1 and 35.2% of the patients. The ANA pattern showed interesting associations with several immune markers that are documented to have significant clinical implications in SLE. For instance, the peripheral, mixed, and speckled patterns were associated with higher ANA titers compared to the other patterns. Further, the peripheral pattern was associated with highest levels of anti-dsDNA and lowest levels of C4, and showed higher levels of CRP and ACL and β2-glycoprotein antibodies, including both IgG and IgM, compared to the other patterns. On the other hand, the mixed pattern was associated with the highest levels of lupus anticoagulant. The potential significance of these associations and their clinical implications are discussed in the light of the updated literature.

### 4.2 Prevalence of Different ANA Patterns

Consistent with our findings, the speckled pattern was the most frequent in an Egyptian cohort of 300 SLE patients, accounting for 79.5% of the tested patients, followed by the homogeneous (11.4%) and the nucleolar (6.8%) patterns (23). In Poland, the ICAP of a series of 260 SLE patients showed speckled patterns to be the most frequent (64.6%), including dense fine, large and coarse speckled (24). Another Swedish study among 219 patients showed relatively comparable patterns with speckled and homogeneous patterns being the most prevalent; however, the homogeneous pattern (54.3%) was more frequent than speckled (22.4%). Mixed homogeneous-speckled pattern ranked third in terms of prevalence accounting for 11.0% (25). By contrast, data from the Systemic Lupus International Collaborating Clinics Cohort, involving 1137 newly diagnosed SLE patients, showed positive ANA among 92.3% of the patients, and majority of the latter had nuclear (77.1%) or mixed nuclear and cytoplasmic and mitotic cell (15.1%) pattern. On the other hand, the prevalence of speckled pattern was marginal accounting for less than 1% of the total patients (26). The heterogeneity of international data regarding the ANA patterns in SLE may be explained by clinical and ethnic disparity. Earlier studies showed several inter-racial differences in SLE expression, namely, both clinical presentation, biological and immunological parameters and disease activity (27–29). This may be consistent with the variance in ANA pattern across nationalities that were found in the present study.

### 4.3 Significance of the Association of ANA Pattern With ANA Titer and Anti-dsDNA

One of the remarkable findings from this study is the significantly higher ANA titers and anti-dsDNA levels that were found in the peripheral pattern compared with the other patterns. On the other hand, the mixed and speckled patterns showed the second highest titers of ANA. Statistically wise, the ANA titer variable was analyzed both as categorical, using chi square test for cross-tabulation, and as an ordinal variable, using a ranked nonparametric test, namely Kruskal–Wallis test; both tests showed high levels of significance. Nonetheless, where this analysis may be limited in power, due to the small size of the peripheral pattern group (N = 10), lower ANA titer in homogeneous pattern was statistically significant compared to speckled and mixed patterns, while the difference between speckled versus mixed patterns was not significant.

ANA is a term that encompasses a range of autoantibodies targeting any of the nuclear constituents, be that a nucleic acid (NA, such as DNA or RNA), a protein or a protein–NA complex. Nonetheless, being a broader designation, the term ANAs is also used to designate autoantibodies targeting other than the nucleus constituents, notably those targeting cytoplasmic proteins such as the anti-ribosomal P (RibP). Furthermore, ANAs share some common features and may have overlapping expressions, which makes them a distinct entity regardless of the antigenic target. As such, immunofluorescence assay using Hep-2 cell line kits represents the key test to characterize ANAs by determining their positivity, staining patterns and titers (19, 30, 31). Besides being considered as quintessential markers in SLE, characterizing ANAs titers and antigenic target is predictive of the clinical manifestations of the disease, its pathogenicity and primary site of activity. Most specifically, anti-ds DNAs, a sub-category of anti-DNA ANAs that bind the double-stranded DNA, have a higher diagnostic value in SLE, and their titers have long been observed to indicate the disease activity and level of organ involvement, notably SLE nephritis (11–14). Other highly specific ANAs for SLE are anti-Smith (anti-Sm) antibodies, which are a subset of anti-RNA antibodies that bind ribosomal proteins and ribosome-containing complexes (32). Both anti-DA and anti-Sm antibodies are considered among the classification criteria of SLE. This is advocated by the new EULAR/ACR consensual classification, which showed a high accuracy with 96% sensitivity and 93% specificity (22). Considering these observations, findings from the present study show significantly higher ANA and anti-dsDNA profiles in the peripheral staining pattern, support the hypothesis that such pattern may be associated with higher disease activity and may be predictive of greater organ damage. Differences across other patterns suggest further clinical significance to ANA pattern and ANA and anti-DNA profiles. Comparable to our findings, the speckled pattern showed the highest profiles of anti-RNA including anti-Sm and anti-snRNP in Swedish LE patients; however, no data for peripheral pattern was reported (25). The same study demonstrated significant clinical association, whereby the speckled pattern was associated with reduced risk of arthritis, immunological disorders and organ damage; whereas the homogeneous pattern was associated with more frequent immunological disorders.

Notwithstanding the new EULAR/ACR criteria and the aforementioned pathological correlations, the clinical use and significance of ANAs and anti-DNA in SLE patients is increasingly challenged by recent clinical trials and experiments, revealing high percentages of SLE patients with negative ANA (15–17). Furthermore, great mystery and uncertainties hover around the pathogenic potentials and binding features of each specific anti-DNA (12, 18). Additionally, the current assays used in ANA and anti-DNA testing present several technical issues that question their reliability and interpretability, notably issues potentially impacting the interaction of synthetic components of each assay with the antibodies of patient (21). This results in erratic performance and variable scope of anti-DNA identification between different assays, which constitute the major drawback delaying consensus regarding the use and significance of ANA and anti-DNA assays in SLE (19, 20). This suggests further investigations are warranted to address this issue, notably by including more patients from rare patterns and using different assays and control groups.

### 4.4 Significance of the Association of ANA Pattern With C3 and C4

The second interesting observation from the present study is the association of ANA pattern with the complement, further emphasizing the peripheral pattern. While no significant association was observed with C3, C4 levels were significantly lower in patients with peripheral pattern. Abnormal levels of C3 and or C4 are indicative for complement consumption, which in SLE is correlated with the disease activity. Notably, higher levels of C4 are associated with higher rate of flares; whereas decreased C3 or C4 levels are significantly associated with organ involvement, especially in the renal subscale of the lupus activity index (33). Other data support that SLE patients with fluctuant levels of complement are at higher risk for lupus glomerulonephritis, with reference to those with constantly low or normal levels (34). In another study investigating the significance of C3 and C4 in antiphospholipid syndrome, approximately, 40% lower levels of C4 (both C4A and C4B) were observed in thrombotic SLE patients with reference to thrombosis-free SLE patients (35).

In the present study, the association of peripheral ANA pattern with lower C4 levels further supports the high potential of the same pattern to induce organ injury, notably renal damage. Consequently, patients having such ANA pattern may require specific attention including a closer monitoring and more adapted treatment protocols to mitigate the higher risk for organ damage. In line with these conclusions, we relate an unpublished case from our department. It consisted of a 24-year-old woman who presented with pericarditis with fever and diagnostic immunology testing, namely, ANA and anti-DsDNA were requested. Results were reported to be ANA 1:80 with peripheral pattern and anti-dsDNA was normal. Although, the diagnosis of SLE was ruled out, a close monitoring was recommended by the consultant diagnostic immunologist. Nevertheless, the patient was lost for follow-up. Six months later, the patient presented to the emergency department for an active SLE with active renal failure.

### 4.5 Significance of the Association of ANA Pattern With Antiphospholipid Antibodies

The last but not the least finding of interest in the present study is the association of peripheral pattern with a higher prevalence of antiphospholipid antibodies (aPLs) ACL and anti-β2 glycoprotein antibodies compared with the other patterns. Some patterns, namely, nucleolar and centromere, showed complete absence of ACL and anti-β2 glycoprotein antibodies. On the other hand, although lupus anticoagulant was positive in 99.4% of the patients, levels were significantly higher in patients with mixed pattern. The positivity rates for the other aPLs, regardless of the ANA pattern, showed ACL (13 and 10.9%) and anti-β2 glycoprotein (21.7 and 8.1%) including IgG and IgM, respectively.

Overall, aPL detection rate in SLE patients is reported to be relatively high, ranging between 30 and 86% depending on the antibody and the study (36–39). However, the detection of aPL is not equivalent to the diagnosis of antiphospholipid syndrome (APS), which was reported to coexist with SLE in less than 10% (40, 41) to 25.4% (37) of the SLE patients. SLE patients fulfilling the criteria of APS have a high risk for mortality and morbidity including cardiovascular and thromboembolic events, notably ischemic stroke, obstetric morbidity (40, 41), besides a multisystem involvement (37). Additionally, APS was observed to be particularly associated with pulmonary involvement in SLE patients (42). On the other hand, a study involving 525 SLE and non-SLE APS patients showed that thrombotic events among SLE-APS patients was associated with a higher positivity rate of lupus anticoagulant (76.3% vs 51.2% p <0.0001) compared with SLE-APS patients without thrombosis, respectively (35). Regarding mortality, a study involving 679 SLE patients showed that those having APS had significantly higher mortality rate than their counterparts, with myocardial infarction being the most frequent cause of death (41).

Nonetheless, beyond the full APS picture, a positive detection of aPLs in SLE is individually associated with high morbidity. A review of meta-analyses and prospective studies showed that aCL is associated with up to 3.7 odd ratio (OR) for pregnancy morbidity and 2.4 to 5.8 OR for other clinical manifestations such as venous thromboembolism, thrombocytopenia, hemolytic anemia, impaired renal function and valvular disease. Likewise, anti-β2GPI and lupus anticoagulant were associated each with up to 8.9 and 3.7 OR for pregnancy morbidity, respectively, and 2.0 to 5.6 OR for previously mentioned clinical manifestations (43). Other data showed a significant correlation of aPL with SLE activity and cognitive dysfunctions (44). Of note, authors of the previous review proposed that a clinically significant aPL should be defined as a “positive (lupus anticoagulant) test based on the guidelines of International Society of Thrombosis and Haemostasis, aCL IgG/IgM ≥40 U, and/or anti-β2GPI IgG/IgM ≥40 U, tested twice at least 12 weeks apart” (43). Further, authors stressed on the cautious interpretation of the results in patients on anticoagulant therapy. The prevalence of clinically significant aPL profile in SLE was estimated to be 20% (45).

### 4.6 Antiphospholipid Antibodies Isotypes

While the positivity rate of IgM in aPLs was lowers than that of IgG in all patients, those with peripheral ANA pattern had comparable, high rates of IgM and IgG. The significance of the aPL antibodies isotype is still a question mark. A study among 796 SLE patients showed that positive IgM anti-β2GPI was associated with a 2.6-fold risk of ischemic attack, while it was associated with a reduced risk for hypertension (OR = 0.54) and renal damage, namely, lupus nephritis (OR = 0.54), persistent proteinuria (OR = 0.19), and renal SLE (OR = 0.58) with reference to negative IgM anti-β2GPI and regardless of the IgG status (46). Authors titled their paper “IgM Anti-ß_2_ Glycoprotein I Is Protective Against Lupus Nephritis and Renal Damage in Systemic Lupus Erythematosus”. An earlier study involving 100 SLE patients showed that positive IgM aCL was predictive for hemolytic anemia and neutropenia with 56 and 84% sensitivity and 80 and 83% specificity, respectively (47). Recently, the pathogenic implications of IgA isotype of aCL and anti-B2GPI have been demonstrated. Notably, a positive association with an increased risk of thrombotic events, especially, when associated with positive lupus anticoagulant. Its prevalence in SLE patients ranged between 2–87% for aCL and 14–100% for anti-B2GPI (47–52).

### 4.7 Limitations

The present study is limited by the retrospective design and the use of immunodiagnostic laboratory data. This explains the lack of a control group and clinical correlations with ANA patterns. Furthermore, multiple comparison analysis was not practicable due to missing data of autoimmune markers in a relatively high proportion of patients as shown in **Table 2**.

### 4.8 Implications and Conclusions

The synthetic indication regarding the clinical implications of our findings, combined with the review of the demonstrated and potential pathogenic roles of the different immune markers and antibody isotypes, suggests that SLE patients with peripheral, speckled and mixed ANA staining patterns have a greater likelihood for more severe disease and organ damage. This supports the potential utility of immunofluorescence assay in further characterizing and or predicting the disease activity for eventual adaptive management strategy. Nevertheless, the present study has a major limitation due to the small size in some ANA pattern groups, notably in peripheral pattern. This impacts the generalizability of the findings notably those concerning this specific pattern.

Given the complexity of SLE and the great number of immunological parameters underlying its pathogenicity, it is reasonable to advocate for ANA patterns testing to enhance our understanding of the disease. In a big data perspective, combined with the forecasted progress in immune and molecular methods, data generated from ANA pattern assays will probably provide precious contribution in the elucidation of the immunological mechanisms and their clinical and therapeutic implications in SLE.

## Data Availability Statement

The data analyzed in this study is subject to the following licenses/restrictions: Data for the present study are available upon request from the author. Requests to access these datasets should be directed to almughales@gmail.com.

## Ethics Statement

The studies involving human participants were reviewed and approved by the Institutional review board of the King Abdulziz University, Jeddah, Saudi Arabia. Written informed consent from the legal guardian/next of kin of the participants was not required to participate in this study in accordance with the national legislation and the institutional requirements.

## Author Contributions

The author declares being the sole contributor in the present paper. He designed the study, collected the data, guided and critically appraised the statistical analysis, and drafted and approved the final version of the manuscript.

## Funding

This study received a grant from the Deanship of Scientific Research of King AbdulAziz University, Jeddah, Saudi Arabia (Grant no KEP-9-140-42).

## Conflict of Interest

The author declares that the research was conducted in the absence of any commercial or financial relationships that could be construed as a potential conflict of interest.

## Publisher’s Note

All claims expressed in this article are solely those of the authors and do not necessarily represent those of their affiliated organizations, or those of the publisher, the editors and the reviewers. Any product that may be evaluated in this article, or claim that may be made by its manufacturer, is not guaranteed or endorsed by the publisher.

## References

[1] Györi N Giannakou I Chatzidionysiou K Magder L van Vollenhoven RF Petri M. Disease Activity Patterns Over Time in Patients With SLE: Analysis of the Hopkins Lupus Cohort. Lupus Sci Med (2017) 4:e000192. doi: 10.1136/lupus-2016-000192 28243457 PMC5307372

[2] Karnal EM Fabian J Carlesso LC Gelinski JMLN. Primary Nutritional Guidance at Systemic Lupus Erythematosus. J Sci Res Rep (2020) 26:10–9. doi: 10.9734/jsrr/2020/v26i530256

[3] Fava A Petri M. Systemic Lupus Erythematosus: Diagnosis and Clinical Management. J Autoimmun (2019) 96:1–13. doi: 10.1016/j.jaut.2018.11.001 30448290 PMC6310637

[4] Gurevitz SL Snyder JA Wessel EK Frey J Williamson BA. Systemic Lupus Erythematosus: A Review of the Disease and Treatment Options. Consult Pharm (2013) 28:110–21. doi: 10.4140/TCP.n.2013.110 23395811

[5] Yaniv G Twig G Shor DB Furer A Sherer Y Mozes O. A Volcanic Explosion of Autoantibodies in Systemic Lupus Erythematosus: A Diversity of 180 Different Antibodies Found in SLE Patients. Autoimmun Rev (2015) 14:75–9. doi: 10.1016/j.autrev.2014.10.003 25449682

[6] Han S Zhuang H Shumyak S Yang L Reeves WH. Mechanisms of Autoantibody Production in Systemic Lupus Erythematosus. Front Immunol (2015) 6:228. doi: 10.3389/fimmu.2015.00228 26029213 PMC4429614

[7] Fattal I Shental N Mevorach D Anaya J-M Livneh A Langevitz P. An Antibody Profile of Systemic Lupus Erythematosus Detected by Antigen Microarray. Immunology (2010) 130:337–43. doi: 10.1111/j.1365-2567.2010.03245.x PMC2913213 20201986

[8] Xu XF Zhang J Cui L Wang YH Yue Y Chi L. The Value of Different Antibodies Detection in Diagnosis of Rheumatism With Uveitis. Zhonghua yi xue za zhi (2017) 97:285–90. doi: 10.3760/cma.j.issn.0376-2491.2017.04.010 28162159

[9] Aringer M Leuchten N Johnson SR. New Criteria for Lupus. Curr Rheumatol Rep (2020) 22:18. doi: 10.1007/s11926-020-00896-6 32405775 PMC7220972

[10] Song XY Huang H Liu YZ Zhao YY Li S Xu ZJ. Coexistence of Sarcoidosis and Primary Sjögren Syndrome: A Clinical Analysis and Literature Review. Zhonghua nei ke za zhi (2017) 56:375–7. doi: 10.3760/cma.j.issn.0578-1426.2017.05.014 28460510

[11] Pisetsky DS. Anti-DNA Antibodies — Quintessential Biomarkers of SLE. Nat Rev Rheumatol (2016) 12:102–10. doi: 10.1038/nrrheum.2015.151 26581343

[12] Rekvig OP. The Anti-DNA Antibody: Origin and Impact, Dogmas and Controversies. Nat Rev Rheumatol (2015) 11:530–40. doi: 10.1038/nrrheum.2015.69 26034836

[13] Ward MM Pisetsky DS Christenson VD. Antidouble Stranded DNA Antibody Assays in Systemic Lupus Erythematosus: Correlations of Longitudinal Antibody Measurements. J Rheum (1989) 16:609–13. 2666655

[14] ter Borg EJ Horst G Hummel EJ Limburg PC Kallenberg CGM. Measurement of Increases in Anti-Double-Stranded DNA Antibody Levels as a Predictor of Disease Exacerbation in Systemic Lupus Erythematosus. Arthritis Rheum (1990) 33:634–43. doi: 10.1002/art.1780330505 2346519

[15] Pisetsky DS Rovin BH Lipsky PE. New Perspectives in Rheumatology: Biomarkers as Entry Criteria for Clinical Trials of New Therapies for Systemic Lupus Erythematosus: The Example of Antinuclear Antibodies and Anti-DNA. Arthritis Rheumatol (2017) 69:487–93. doi: 10.1002/art.40008 27899010

[16] Furie R Petri M Zamani O Cervera R Wallace DJ Tegzová D. Randomized, Placebo-Controlled Study of Belimumab, a Monoclonal Antibody That Inhibits B Lymphocyte Stimulator, in Patients With Systemic Lupus Erythematosus. Arthritis Rheum (2011) 63:3918–30. doi: 10.1002/art.30613 PMC5007058 22127708

[17] Wallace DJ Stohl W Furie RA Lisse JR McKay JD Merrill JT. Randomized, Double-Blind, Placebo-Controlled, Dose-Ranging Study of Belimumab in Patients With Active Systemic Lupus Erythematosus. Arthritis Rheum (2009) 61:1168–78. doi: 10.1002/art.24699 PMC2758229 19714604

[18] Rekvig OP van der Vlag J Seredkina N. Review: Antinucleosome Antibodies: A Critical Reflection on Their Specificities and Diagnostic Impact. Arthritis Rheumatol (2014) 66:1061–9. doi: 10.1002/art.38365 24470458

[19] Pisetsky DS. Antinuclear Antibody Testing — Misunderstood or Misbegotten? Nat Rev Rheumatol (2017) 13:495–502. doi: 10.1038/nrrheum.2017.74 28541299

[20] Pisetsky DS Bossuyt X Meroni PL. ANA as an Entry Criterion for the Classification of SLE. Autoimmun Rev (2019) 18:102400. doi: 10.1016/j.autrev.2019.102400 31639513

[21] Pisetsky DS Lipsky PE. New Insights Into the Role of Antinuclear Antibodies in Systemic Lupus Erythematosus. Nat Rev Rheumatol (2020) 16:565–79. doi: 10.1038/s41584-020-0480-7 PMC8456518 32884126

[22] Aringer M Costenbader K Daikh D Brinks R Mosca M Ramsey-Goldman R. European League Against Rheumatism/American College of Rheumatology Classification Criteria for Systemic Lupus Erythematosus. Arthritis Rheumatol (2019) 71:1400–12. doi: 10.1002/art.40930 PMC6827566 31385462

[23] Elamir A Farid A Amin E Hassan H Maged M Aref A. Anti-Nuclear Antibody (ANA) Patterns in Egyptian Systemic Lupus Erythematosus. J Egypt Soc Parasitol (2019) 49:451–4. doi: 10.21608/JESP.2019.68190

[24] Krzemień P Kasperczyk S Banach M Kasperczyk A Dobrakowski M Tomasik T. Analysis of the Impact of Sex and Age on the Variation in the Prevalence of Antinuclear Autoantibodies in Polish Population: A Nationwide Observational, Cross-Sectional Study. Rheumatol Int (2021) 42:261–71. doi: 10.1007/s00296-021-05033-9 PMC8800880 34755204

[25] Frodlund M Dahlström Ö Kastbom A Skogh T Sjöwall C. Associations Between Antinuclear Antibody Staining Patterns and Clinical Features of Systemic Lupus Erythematosus: Analysis of a Regional Swedish Register. BMJ Open (2013) 3:e003608. doi: 10.1136/bmjopen-2013-003608 PMC3808756 24163206

[26] Choi MY Clarke AE Pierre Y Hanly JG Urowitz MB Romero-Diaz J. Antinuclear Antibody–Negative Systemic Lupus Erythematosus in an International Inception Cohort. Arthritis Care Res (2019) 71:893–902. st. doi: 10.1002/acr.23712 PMC7268889 30044551

[27] Wang F Wang CL Tan CT Manivasagar M. Systemic Lupus Erythematosus in Malaysia: A Study of 539 Patients and Comparison of Prevalence and Disease Expression in Different Racial and Gender Groups. Lupus (1997) 6:248–53. doi: 10.1177/096120339700600306 9104731

[28] Alarcón GS Friedman AW Straaton K v Moulds JM Lisse J Bastian HM. Systemic Lupus Erythematosus in Three Ethnic Groups: III a Comparison of Characteristics Early in the Natural History of the LUMINA Cohort. Lupus (1999) 8:197–209. doi: 10.1191/096120399678847704 10342712

[29] Jurencák R Fritzler M Tyrrell P Hiraki L Benseler S Silverman E. Autoantibodies in Pediatric Systemic Lupus Erythematosus: Ethnic Grouping, Cluster Analysis, and Clinical Correlations. J Rheumatol (2009) 36:416–21. doi: 10.3899/jrheum.080588 19208567

[30] Agmon-Levin N Damoiseaux J Kallenberg C Sack U Witte T Herold M. International Recommendations for the Assessment of Autoantibodies to Cellular Antigens Referred to as Anti-Nuclear Antibodies. Ann Rheum Dis (2014) 73:17–23. doi: 10.1136/annrheumdis-2013-203863 24126457

[31] Choi MY FitzPatrick RD Buhler K Mahler M Fritzler MJ. A Review and Meta-Analysis of Anti-Ribosomal P Autoantibodies in Systemic Lupus Erythematosus. Autoimmun Rev (2020) 19:102463. doi: 10.1016/j.autrev.2020.102463 31927088

[32] Ahn SS Jung SM Yoo J Lee S-W Song JJ Park Y-B. Anti-Smith Antibody is Associated With Disease Activity in Patients With New-Onset Systemic Lupus Erythematosus. Rheumatol Int (2019) 39:1937–44. doi: 10.1007/s00296-019-04445-y 31552434

[33] Ho A Barr SG Magder LS Petri M. A Decrease in Complement is Associated With Increased Renal and Hematologic Activity in Patients With Systemic Lupus Erythematosus. Arthritis Rheum (2001) 44:2350–7. doi: 10.1002/1529-0131(200110)44:10<2350::aid-art398>3.0.co;2-a 11665976

[34] Gandino IJ Scolnik M Bertiller E Scaglioni V Catoggio LJ Soriano ER. Complement Levels and Risk of Organ Involvement in Patients With Systemic Lupus Erythematosus. Lupus Sci Med (2017) 4:e000209. doi: 10.1136/lupus-2017-000209 29259790 PMC5729297

[35] Savelli SL Roubey RAS Kitzmiller KJ Zhou D Nagaraja HN Mulvihill E. Opposite Profiles of Complement in Antiphospholipid Syndrome (APS) and Systemic Lupus Erythematosus (SLE) Among Patients With Antiphospholipid Antibodies (Apl). Front Immunol (2019) 10:885. doi: 10.3389/fimmu.2019.00885 31134052 PMC6514053

[36] Petri M. Epidemiology of the Antiphospholipid Antibody Syndrome. J Autoimmun (2000) 15:145–51. doi: 10.1006/jaut.2000.0409 10968901

[37] Singh NK Agrawal A Singh MN Kumar V Godhra M Gupta A. Prevalence and Pattern of Antiphospholipid Antibody Syndrome in a Hospital Based Longitudinal Study of 193 Patients of Systemic Lupus Erythematosus. J Assoc Physicians India (2013) 61:623–6. 24772699

[38] Noori AS Jawad AM Jassim NA and Gorial FI. Prevalence of Antiphospholipid Antibodies in Sample of Iraqi Patients With Systemic Lupus Erythematosus: A Cross Sectional Study. Am J Clin Med Res (2013) 1:61–4. doi: 10.12691/ajcmr-1-4-4

[39] Marchetti T Ribi C Perneger T Trendelenburg M Huynh-Do U de Moerloose P. Prevalence, Persistence and Clinical Correlations of Classic and Novel Antiphospholipid Antibodies in Systemic Lupus Erythematosus. Rheumatology (2018) 57:1350–7. doi: 10.1093/rheumatology/key095 29672737

[40] Franco J-S Molano-González N Rodríguez-Jiménez M Acosta-Ampudia Y Mantilla RD Amaya-Amaya J. The Coexistence of Antiphospholipid Syndrome and Systemic Lupus Erythematosus in Colombians. PloS One (2014) 9:e110242. doi: 10.1371/journal.pone.0110242 25343509 PMC4208791

[41] Mok CC Chan PT Ho LY Yu KL To CH. Prevalence of the Antiphospholipid Syndrome and its Effect on Survival in 679 Chinese Patients With Systemic Lupus Erythematosus. Medicine (2013) 92:217–22. doi: 10.1097/MD.0b013e31829cae47 PMC4553973 23793109

[42] Kokosi M Lams B Agarwal S. Systemic Lupus Erythematosus and Antiphospholipid Antibody Syndrome. Clin Chest Med (2019) 40:519–29. doi: 10.1016/j.ccm.2019.06.001 31376888

[43] Unlu O Zuily S Erkan D. The Clinical Significance of Antiphospholipid Antibodies in Systemic Lupus Erythematosus. Eur J Rheumatol (2016) 3:75–84. doi: 10.5152/eurjrheum.2015.0085 27708976 PMC5042235

[44] Conti F Alessandri C Perricone C Scrivo R Rezai S Ceccarelli F. Neurocognitive Dysfunction in Systemic Lupus Erythematosus: Association With Antiphospholipid Antibodies, Disease Activity and Chronic Damage. PloS One (2012) 7:e33824. doi: 10.1371/journal.pone.0033824 22461897 PMC3312889

[45] Taraborelli M Leuenberger L Zhang W Tincani A Salmon J Erkan D. The Effect of Clinically Significant Antiphospholipid Antibody Positivity on Organ Damage in Systemic Lupus Erythematosus. Abstract 18. In: 2014 ACR/ARHP Annual Meeting (November 14–19). Boston, MA: American College of Rheumatology (2014). p. 66. Available at: https://acrabstracts.org/abstract/the-effect-of-clinically-significant-antiphospholipid-antibody-positivity-on-organ-damage-in-systemic-lupus-erythematosus/.

[46] Mehrani T Petri M. Igm Anti-ß 2 Glycoprotein I is Protective Against Lupus Nephritis and Renal Damage in Systemic Lupus Erythematosus. J Rheumatol (2011) 38:450–3. doi: 10.3899/jrheum.100650 21123325

[47] Cervera R Font J Lopez-Soto A Casals F Pallares L Bove A. Isotype Distribution of Anticardiolipin Antibodies in Systemic Lupus Erythematosus: Prospective Analysis of a Series of 100 Patients. Ann Rheum Dis (1990) 49:109–13. doi: 10.1136/ard.49.2.109 PMC1003989 2107799

[48] Lopez LR Santos ME Espinoza LR la Rosa FG. Clinical Significance of Immunoglobulin a Versus Immunoglobulins G and M Anti-Cardiolipin Antibodies in Patients With Systemic Lupus Erythematosus: Correlation With Thrombosis, Thrombocytopenia, and Recurrent Abortion. Am J Clin Pathol (1992) 98:449–54. doi: 10.1093/ajcp/98.4.449 1415024

[49] Meijide H Sciascia S Sanna G Khamashta MA Bertolaccini ML. The Clinical Relevance of Iga Anticardiolipin and Iga Anti-β2 Glycoprotein I Antiphospholipid Antibodies. Autoimmun Rev (2013) 12:421–5. doi: 10.1016/j.autrev.2012.08.002 22951216

[50] Akhter E Shums Z Norman Gl Binder W Fang H Petri M. Utility of Antiphosphatidylserine/Prothrombin and Iga Antiphospholipid Assays in Systemic Lupus Erythematosus. J Rheum (2013) 40:282–6. doi: 10.3899/jrheum.120084 PMC3605900 23378459

[51] Andreoli L Fredi M Nalli C Piantoni S Reggia R Dall’Ara F. Clinical Significance of Iga Anti-Cardiolipin and Iga Anti-β2glycoprotein I Antibodies. Curr Rheumatol Rep (2013) 15:343. doi: 10.1007/s11926-013-0343-1 23754504

[52] Demir S Li J Magder LS Petri M. Antiphospholipid Patterns Predict Risk of Thrombosis in Systemic Lupus Erythematosus. Rheumatology (2021) 60:3770–7. doi: 10.1093/rheumatology/keaa857 PMC8328497 33331921

